# BRCA1/2 and CHEK2 Pathogenic Variants in Urological Cancers: A Portuguese Single-Center Experience

**DOI:** 10.7759/cureus.105002

**Published:** 2026-03-10

**Authors:** Margarida Quinto Pereira, Isália Miguel, Sofia Fragoso, Teresa Duarte, Sidónia Santos, Paula Rodrigues, Carolina Pereira, Fátima Vaz

**Affiliations:** 1 Medical Oncology Department, Instituto Português de Oncologia de Lisboa Francisco Gentil, Lisbon, PRT; 2 Molecular Pathobiology Research Unit, Instituto Português de Oncologia de Lisboa Francisco Gentil, Lisbon, PRT; 3 Familial Cancer Clinic Unit, Instituto Português de Oncologia de Lisboa Francisco Gentil, Lisbon, PRT

**Keywords:** brca1/2 genes, chek2 gene, genetic testing, hereditary cancer, urological cancer

## Abstract

Background

Germline pathogenic variants (gPVs) in *BRCA1*, *BRCA2*, and *CHEK2* are well‑established cancer predisposition factors of prostate cancer. However, their contribution across the full spectrum of urological cancers remains insufficiently characterized.

The primary objective was to describe the spectrum and outcomes of urological cancers in *BRCA1/2* and *CHEK2* gPV carriers, while secondary objectives were to assess overall survival (OS) and compare age at diagnosis, stage, and survival outcome by gene affected.

Methods

This is a retrospective descriptive study including patients diagnosed with urological cancers and testing positive for gPVs in *BRCA1*, *BRCA2*, or *CHEK2*, identified at the Hereditary Cancer Risk Clinic of the Instituto Português de Oncologia de Lisboa Francisco Gentil (IPOLFG). Familial and individual files were systematically reviewed to identify individuals with urological malignancies carrying gPVs. Descriptive statistics were used to summarize clinicopathologic characteristics. Survival outcomes were estimated using the Kaplan-Meier method and log-rank test for comparisons between groups, with p<0.05 considered statistically significant.

Results

A total of 968 *BRCA1/2* or *CHEK2* families were identified, of which 47 included 50 patients with urological cancer and a gPV in the genes of interest. Among *BRCA1/2* carriers, *BRCA2* was the most frequently affected gene (37 patients), with the *BRCA2* Portuguese founder variant (c.156_157insAlu) identified in 23.1% of cases.

Among prostate cancer patients carrying *BRCA1/2* gPVs, all individuals with high-grade or metastatic disease carried *BRCA2 *gPVs. These patients were diagnosed at a younger age than either what is expected from the general population or patients with *BRCA1*-associated prostate cancer in this study. Also, *BRCA2 *prostate cancer patients were more frequently diagnosed with other cancers, with male breast cancer being the most frequent (32%). Regarding family history, breast cancer was the most common malignancy observed (71% in *BRCA1 *and 74% in *BRCA2 *families), followed by other cases of prostate cancer (42% in *BRCA1 *vs. 34% in *BRCA2*).

The median OS among prostate cancer patients with advanced disease was 38 months (95% CI: 5.6-70.3), with no statistically significant difference between *BRCA1 *and *BRCA2 *carriers (p=0.408).

All renal cancer patients with *BRCA *gPVs were female, had a personal history of breast cancer, and presented clear cell histology. Among urothelial cancer patients, one carried a *BRCA1 *gPV, and six carried *BRCA2 *gPVs. Most presented with non-muscle‑invasive bladder cancer (71.4%), except for two *BRCA2 *carriers who had advanced disease.

*CHEK2*‑associated urological cancers included renal cancer (n=1) and prostate cancer (n=5), all of which showed favorable outcomes. No testicular cancers were identified.

Conclusion

This study highlights the heterogeneous spectrum of urological cancers associated with *BRCA1*, *BRCA2*, and *CHEK2 *gPVs in a Portuguese cohort, including the role of the Portuguese founder variant. While prostate cancer is the most frequent urological cancer in these families, increasing access to and awareness of genetic testing may better inform future studies about these cancer phenotypes. We reinforce that the systematic assessment of personal and family cancer history in these patients may assist in identifying individuals who could benefit from genetic evaluation and tailored surveillance strategies for carriers and their families.

## Introduction

Breast cancer susceptibility genes 1 and 2 (*BRCA1* and *BRCA2*) are high‑penetrance tumor suppressor genes that play a central role in homologous recombination DNA repair. Germline pathogenic variants (gPVs) in these genes are well‑established risk factors for hereditary breast and ovarian cancer and have also been associated with an increased risk of several urological malignancies, particularly prostate cancer [1,2].

Men with *BRCA2* gPVs have a substantially elevated lifetime risk of prostate cancer and are more likely to present with aggressive, high‑grade tumors and higher prostate cancer‑specific mortality. *BRCA1* variants also confer an increased prostate cancer risk, although the effect appears smaller than that of *BRCA2* [2-4].

Regarding germline genetic testing in prostate cancer, recommendations were first introduced into the National Comprehensive Cancer Network (NCCN) hereditary cancer risk guidelines in the early 2010s and have since undergone steady expansion in clinical indications. Much of the initial evidence supporting these recommendations was driven by studies demonstrating a stronger and more consistent association with *BRCA2* gPV [5]. Consequently, hereditary testing frameworks have historically placed greater emphasis on *BRCA2*, while the risk association with *BRCA1* has remained comparatively weaker and more variable.

Over subsequent years, NCCN recommendations expanded substantially and now endorse germline genetic testing for other homologous recombination repair genes, including *ATM*, *BRCA1*, *BRCA2*, *CHEK2*, *HOXB13*, *PALB2*, and *TP53*, in patients with a personal history of metastatic or node‑positive prostate cancer, as well as in those with high‑risk localized disease [6]. This broadening of testing criteria has also been accelerated by the therapeutic implications of gPV, particularly with the introduction of poly(ADP‑ribose) polymerase inhibitors (PARP inhibitors), which have made gPVs relevant for clinical management by demonstrating predictive value for response to PARP inhibitors and platinum‑based chemotherapy [7-9].

Regarding prostate cancer surveillance in *BRCA1/2* carriers, available evidence indicates a significantly higher detection rate of clinically significant prostate cancer among *BRCA2 *carriers, supporting the implementation of targeted screening strategies in this population [10,11]. Accordingly, NCCN currently recommends annual prostate cancer screening with prostate-specific antigen (PSA) testing for *BRCA2 *mutation carriers, whereas screening for *BRCA1 *carriers is suggested only for consideration, reflecting the comparatively less consistent risk association [6]. These developments highlight the need to identify carriers of *BRCA1/2 *gPVs and to define which patients with urological cancers should undergo genetic testing, as well as the optimal timing of such testing within their disease course.

Although *BRCA1/2 *gPVs are not traditionally recognized as major risk factors for bladder cancer, emerging evidence suggests a possible association in selected subgroups. Some studies report a higher risk of bladder cancer among female *BRCA1/2 *carriers [12] and link specific *BRCA2 *gPVs to urinary tract malignancies [13]. Furthermore, data from The Cancer Genome Atlas have identified *BRCA2 *gPV in approximately 13% of urothelial carcinomas, raising questions about their biological and clinical relevance [14].

*CHEK2 *is a moderate‑penetrance tumor suppressor gene encoding a checkpoint kinase involved in the DNA damage response. Pathogenic *CHEK2 *variants are most strongly associated with breast cancer, but accumulating evidence implicates *CHEK2 *in a broader tumor spectrum, including prostate and other urological cancers [15-17]. Certain *CHEK2 *variants have been associated with an increased risk of bladder cancer, renal cell carcinoma, and testicular germ cell tumors, although risk estimates remain heterogeneous and clinical management guidelines are still evolving [5,14,18].

Improving the detection of gPVs has the potential to expand access to genetic counselling, tailored cancer screening, and targeted therapies for patients with urological malignancies and their families. Recognition of new hereditary cancer entities, or improved characterization of existing associations, relies on studying at‑risk populations and integrating genomic data into routine clinical practice.

The primary objective of this study is to descriptively characterize patients with urological cancer and carriers of gPVs, followed at a Hereditary Cancer Risk Clinic, specifically regarding gene affected, tumor histology, age at diagnosis, and family and personal cancer history.

The secondary objectives were as follows: (1) to estimate the median overall survival (mOS) for each tumor type; (2) to compare age at diagnosis and stage at presentation, according to the affected gene among *BRCA1/2* carriers; and (3) to perform a strictly exploratory comparison of survival among patients with prostate cancer between *BRCA1 *and *BRCA2 *carriers.

## Materials and methods

Study design and population

This retrospective, descriptive, single-center study was conducted at the Hereditary Cancer Risk Clinic of the Instituto Português de Oncologia de Lisboa Francisco Gentil (IPOLFG), Lisbon, Portugal. We included patients with urological cancers (renal, urothelial, prostate, and testicular) who carried pathogenic or likely pathogenic variants in *BRCA1*, *BRCA2*, or *CHEK2* and who belonged to families followed at the IPOLFG Familial Risk Clinic. Eligible patients were identified from the establishment of the Breast-Ovarian-Prostate Clinic (December 1999) through December 31, 2024.

Patients were referred to the Hereditary Cancer Risk Clinic through established institutional pathways in accordance with contemporaneous clinical risk-based guidelines for hereditary cancer genetic testing. Eligibility for testing was determined through a structured risk assessment, incorporating personal cancer history (including tumor stage and risk category), age at diagnosis, tumor characteristics, family history suggestive of hereditary predisposition (e.g., multiple affected relatives or early-onset disease), and the presence of a known familial pathogenic variant. Self-referral was also permitted; however, genetic testing was only offered if individuals met the same standardized risk-based eligibility criteria described before.

Given the extended study period, testing indications evolved in parallel with updates to clinical risk guidelines, which progressively broadened eligibility criteria over time. Therefore, inclusion in the present study reflects the applicable risk-based testing criteria in place at the time of referral and genetic evaluation.

Families carrying pathogenic or likely pathogenic variants in *BRCA1*, *BRCA2*, or *CHEK2* were identified. These families were systematically screened for members with a diagnosis of urological cancer. All eligible patients meeting the predefined inclusion criteria during the study period were included consecutively, without sampling.

Inclusion criteria included patients with (1) confirmed diagnosis of urological cancer; (2) presence of a gPV in *BRCA1*, *BRCA2*, or *CHEK2*; and (3) follow-up at the IPOLFG Familial Risk Clinic during the study period. Exclusion criteria were as follows: (1) insufficient clinical documentation to confirm oncologic diagnosis or (2) absence of confirmed germline genetic testing results.

**Figure 1:**
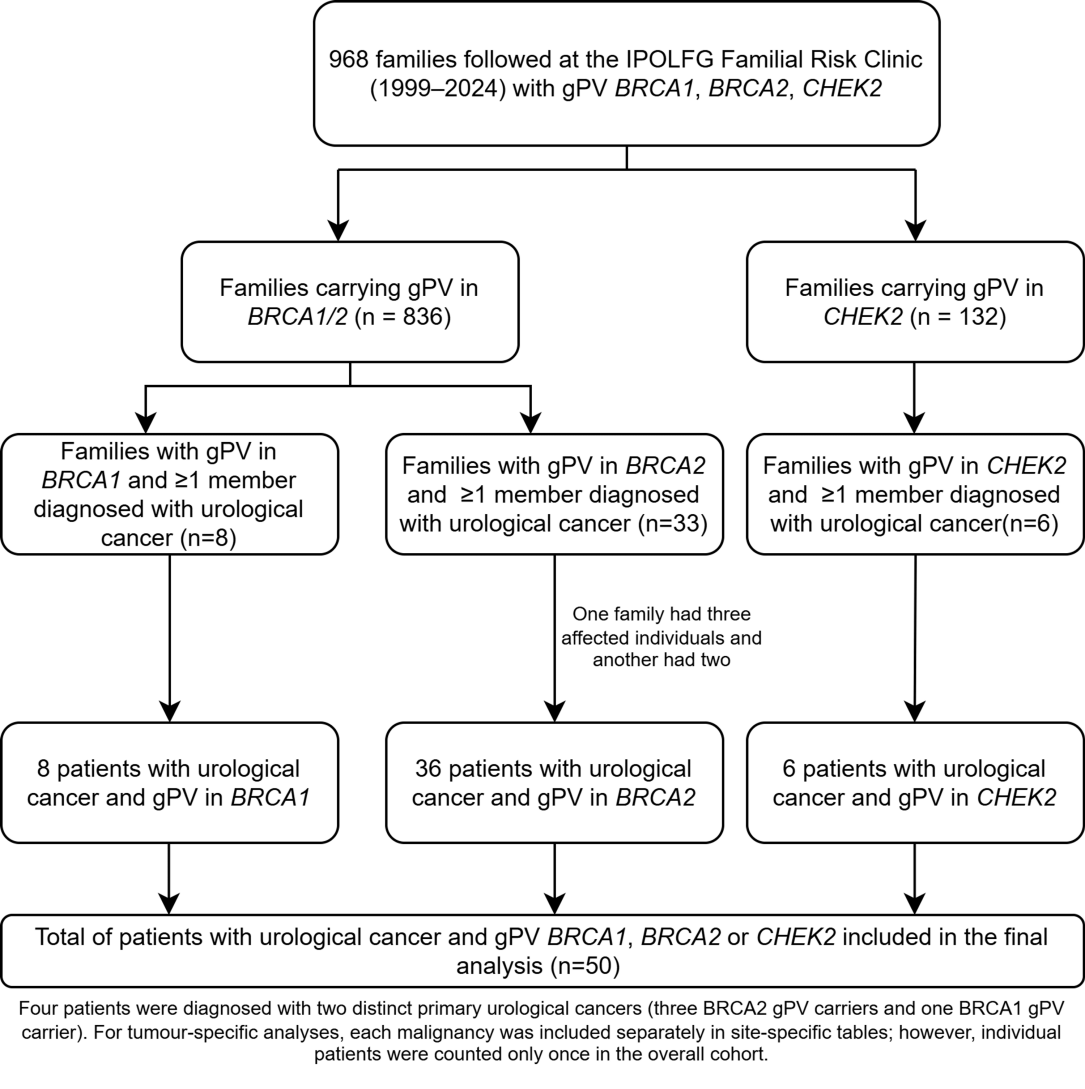
STROBE-style flow diagram of patient inclusion STROBE: Strengthening the Reporting of Observational Studies in Epidemiology; IPOLFG: Instituto Português de Oncologia de Lisboa Francisco Gentil; gPV: germline pathogenic variant

A Strengthening the Reporting of Observational Studies in Epidemiology (STROBE)-style flow diagram summarizing the selection process and the number of families and patients included at each stage is presented in Figure 1.

Data collection

Clinical data were retrospectively extracted from paper and electronic medical records.

Collected variables included the following: genetic data: *BRCA1/2* and *CHEK2* mutational status, affected gene, specific pathogenic variant, and type; demographic and histopathologic data: sex, age at diagnosis, histopathological subtype and initial stage at diagnosis, and smoking history if available; treatment data: type of treatment including surgery, radiotherapy, or systemic therapy; survival data: date of diagnosis, date of death from any cause, and date of last follow-up; and familial and personal history data: personal history of additional other diagnosis of cancer and familial history of cancer.

Variants were named according to ClinGen Expert Panel Specifications to the American College of Medical Genetics and Genomics (ACMG)/Association for Molecular Pathology (AMP) Variant Interpretation Guidelines for *BRCA1*, *BRCA2*, and *CHEK2*. Pathogenic and likely pathogenic variants were both denoted as PV. Variant classification was based on the most up-to-date interpretation available at the time of data extraction. Variants that were newly classified as pathogenic or likely pathogenic during the study period were included in the analysis, even if this classification occurred after the initial clinical evaluation.

The c.1036C>T variant in the *CHEK2* gene was reclassified during the study period, in 2024, following the accumulation of additional evidence, from a variant of unknown significance to likely pathogenic, and was therefore included in this analysis. No other variants were reclassified during the study period.

The initial stage at diagnosis was defined as localized if the tumor was confined to the primary organ, with or without regional lymph node involvement, and metastatic disease was defined as the presence of distant metastases. Histological and staging information was obtained from the original oncologic diagnosis records.

Smoking history was considered positive in individuals who were current or former smokers and negative in those who had never smoked.

Cancer diagnoses were verified through a review of institutional medical records, pathology reports, and, when applicable, documented external clinical reports.

Missing data

The extent of missing data was assessed for all study variables. Smoking status was unavailable in 40% of cases. There were no other variables with missing data. Descriptive analyses were performed using available data, and percentages were calculated based on non-missing observations. No imputation procedures were performed due to the retrospective design and limited sample size.

Molecular diagnosis

Hereditary breast and ovarian cancer testing included the detection of single-nucleotide variants (SNVs) and copy number variants (CNVs) in the *BRCA1 *and *BRCA2 *genes, as well as targeted screening for the Portuguese *BRCA2 *founder variant c.156_157insAlu.* BRCA1/2* testing was performed according to the standard methodologies implemented at the time of analysis.

Between 1999 and 2014, *BRCA1/2* testing was performed using conformation-sensitive gel electrophoresis (CSGE) or conformation-sensitive capillary electrophoresis (CSCE), followed by Sanger sequencing. The Portuguese *BRCA2 *founder variant was screened using a specific polymerase chain reaction (PCR)-based assay, and large deletions and insertions in *BRCA1 *and *BRCA2* were assessed by multiplex ligation-dependent probe amplification (MRC Holland, Amsterdam, Netherlands).

From 2014 to 2018, next-generation sequencing (NGS) was performed using either the TruSight Cancer Sequencing Panel by Illumina (San Diego, California, United States) (including *BRCA1, BRCA2, TP53, PTEN, CDH1, ATM, BRIP1, BLM, BAP1, CHEK2, PALB2, RAD51C, *and *RAD51D*) or the BRCA Hereditary Cancer MASTR™ (*BRCA1 *and *BRCA2*) assay kit by Genycell (Granada, Spain) on a MiSeq platform by Illumina. All pathogenic and likely pathogenic variants identified by NGS were confirmed by Sanger sequencing, while multiplex ligation-dependent probe amplification was used to detect CNVs and the Portuguese founder variant in *BRCA2*.

Since 2018, hereditary breast and ovarian cancer testing has been performed exclusively using multigene panels (including *BRCA1, BRCA2, PALB2, ATM, BARD1, BRIP1, CDH1, CHEK2, PTEN, RAD51C, RAD51D, *and *TP53 *genes) such as Hereditary OncoKit DX combined with DataGenomics software (both by Genycell), enabling the integrated detection of SNVs, CNVs, and the *BRCA2 *founder variant.

Statistical analysis

Statistical analyses were performed using IBM SPSS Statistics for Windows, Version 20.0 (IBM Corp., Armonk, New York, United States). Descriptive statistics were used to summarize patient characteristics and treatments. Continuous variables were reported as medians with ranges and categorical variables as counts and percentages. Group differences were assessed using χ² or Fisher's exact tests, as appropriate. A two-sided p-value of <0.05 was considered statistically significant.

The mOS was defined as the time from the date of diagnosis to death from any cause or last follow-up. Patients who were alive at the last follow-up were censored at that date for mOS analysis. Survival outcomes were estimated using the Kaplan-Meier method, and comparisons between groups were performed using the log-rank test. A p-value of <0.05 was considered statistically significant.

Ethical considerations

The study was conducted in accordance with the Declaration of Helsinki, the General Data Protection Regulation (Regulation EU 2016/679), and national legislation. Data were pseudo-anonymized, with identifying information removed and stored in password-protected databases accessible only to study investigators.

The protocol was approved by the IPOLFG Research and Ethics Committee, which granted a waiver of informed consent because the study involved no intervention and no added risk and relied on previously collected data. In addition, at the IPOLFG Familial Risk Clinic, when patients consent to genetic testing, they are also given the option to participate in other studies approved by the IPOLFG Research and Ethics Committee. Accordingly, only individuals who had provided prior consent, follow-up at the Familial Risk Clinic, and agreement to participation in studies using aggregated data were included. Compliance with Law 21/2014 was ensured.

## Results

We assessed 968 families, of which 47 included patients with urological cancer carrying a gPV in one of the previously described genes, comprising a total of 50 affected individuals. Four patients had a personal history of two distinct urological cancers: three of them were *BRCA2 *gPV carriers, and one was a *BRCA1 *gPV carrier. In addition, one *BRCA2* gPV-carrying family included three relatives with urological cancers, while another had two relatives each affected by urological cancer.

*BRCA1/2* carriers

A total of 836 families with *BRCA1/2* gPV were evaluated. Among these, 41 families (4.9%) included patients with urological malignancies. Across the affected individuals, 37 were diagnosed with prostate cancer, four with renal cancer, and seven with urothelial cancer. No cases of testicular cancer were identified. Regarding the affected gene, the majority (81.8%) carried a *BRCA2 *gPV.

Patients with *BRCA1/2* gPV and prostate cancer

A total of 37 patients from 35 families carrying *BRCA1/2 *gPVs and diagnosed with prostate cancer were evaluated. The affected gene, specific pathogenic variant, age at diagnosis, Gleason score, personal history of cancer, and family cancer history for each patient are summarized in Table 1.

**Table 1:** Prostate cancer in BRCA1/2 carriers Age at diagnosis in years. Gleason score based on the 2005 International Society of Urological Pathology (ISUP) Consensus Conference on Gleason Grading of Prostatic Carcinoma [19]. BCC: basal cell carcinoma; SCC: squamous cell carcinoma; CNS: central nervous system; NA: not available

Gene	Variant	Age at diagnosis	Gleason score	Personal history of other cancers	Familial history
BRCA1	c.5099del	72	G7	-	Breast, prostate
	c.211A>G	77	G6	BCC	Breast, pancreas
	c.1121_1123delCACinsT	78	G7	-	Breast
	c.5266dup	78	G7	Bladder carcinoma	Melanoma, ovarian
	c.5431C>A	56	G7	-	Breast
	c.5266dup	75	G7	Pancreas	Prostate
	c.3331_3334del	65	G7	-	Breast, ovarian, prostate, gastric
BRCA2	c.156_157insAlu	64	G6	0	Breast
	c.156_157insAlu	59	G7	0	Breast, ovarian
	c.156_157insAlu	51	G6	0	Melanoma, lung, breast
	c.156_157insAlu	73	G6	Biliary tract, urothelial	Breast
	c.156_157insAlu	59	G9	0	Colorectal, breast, uterine
	c.156_157insAlu	62	G6	Breast	Breast, prostate
	c.156_157insAlu	68	G6	Cutaneous SCC	Prostate, pancreas, colorectal, breast
	c.156_157insAlu	61	G8	Breast, urothelial	Gastric, pancreas, breast
	c.9097dup	65	G7	Breast cancer, bladder carcinoma	Breast, esophageal
	IVS9+1G>A	48	G6	-	Melanoma, pancreas, prostate
	IVS9+1G>A	76	G6	-	Gastric, colorectal, pancreas
	c.2808_2811del	69	G7	Hepatocarcinoma, colorectal, breast	Colorectal, breast
	c.6676del	73	G8	Breast, BCC	Breast, colorectal, pancreas
	c.7258_7259dup	77	G7	-	Breast, gastric
	c.1588A>T	53	G8	-	Breast, prostate, pancreas
	c.1588A>T	54	G7	-	Breast, prostate, pancreas
	c.1588A>T	71	G6	Multiple myeloma	Breast, prostate, pancreas
	c.2808_2811del	57	G6	BCC	Breast, gastric
	c.67+2T>C	75	NA	-	Thyroid, prostate
	c.4558dup	61	G7	BCC	Dysgerminoma, prostate, cervical cancer
	c.6405_6409del	73	NA	-	Breast
	c.712G>T	61	G8	-	Breast
	c.658_659del	68	G8	Breast	Colorectal
	c.658_659del	70	G9	Colorectal	Colorectal, breast, pancreas
	c.7259_7262dup	51	G7	-	Melanoma, prostate
	c.9815del	43	G7	-	CNS, prostate, colorectal, breast, ovarian
	c.6468_6469del	77	G9	-	Breast
	c.3455T>G	57	G7	-	Breast
	c.8488-1G>A	57	G7	-	CNS, bladder, larynx, colorectal
	c.1056_1058del	48	G6	-	Breast, thymic, prostate

The median age at diagnosis was 75 years (IQR 65-78) for patients with a *BRCA1 *gPV and 61 years (IQR 57-73) for those with a *BRCA2 *gPV.

Five patients (13.5%) presented with metastatic disease at diagnosis. Most patients presented with a Gleason score of 6 (3+3) or 7 (3+4), with 11 patients in each group. A Gleason score of 7 (4+3) was observed in five patients, while five patients had a Gleason score of 8 (4+4). Higher-grade disease was less frequent, with a Gleason score of 9 identified in three patients, including one patient with a score of 5+4 and two patients with a score of 4+5. Gleason score information was unavailable for two patients. All patients with higher-grade or metastatic disease carried a *BRCA2 *gPV, whereas only two patients with a *BRCA1 *gPV had a Gleason score of 7 (4+3).

Smoking history was only reported in 24 patients, with 16 having reported a history of smoking and eight non-smokers.

Regarding personal cancer history, prior cancers were observed in 40% of *BRCA2 *gPV patients and were more diverse, with breast cancer being the most common and occurring exclusively in this group (six patients). Five *BRCA2 *carriers had a history of more than one additional malignancy. Among *BRCA1 *carriers, personal history of other malignancies (in 42.8%of *BRCA1* gPV) consisted predominantly of non-breast cancers.

When considering family history, among *BRCA1 *families, breast cancer was the most frequently reported malignancy (71% of *BRCA1* gPV), followed by prostate cancer (42%). In *BRCA2 *families, breast cancer was also the most prevalent familial malignancy, reported in 21 families (72.4%), followed by prostate cancer, which was reported in eight families (27.6%). *BRCA2 *families exhibited a broader spectrum of associated malignancies, including pancreatic, colorectal, and gastric cancers, as well as melanoma.

With respect to treatment modalities in the setting of localized disease, among *BRCA1 *carriers, five patients (71%) underwent radiotherapy followed by adjuvant hormonal therapy, one patient (14%) received radiotherapy alone, and one patient (14%) received hormonal therapy alone. Among *BRCA2 *carriers, 13 patients (52%) were treated with radiotherapy followed by adjuvant hormonal therapy; five patients (20%) received radiotherapy alone; three patients (12%) underwent surgery followed by adjuvant hormonal therapy; two patients (8%) were treated with surgery alone; one patient (4%) received hormonal therapy without local treatment; and one patient was managed with active surveillance. Among patients with stage IV prostate cancer, three were treated with chemotherapy, and two received treatment with novel hormonal agents.

The median follow-up was 7±4.9 years. The mOS was 167 months (95% CI: 131.0-202.9) in patients with localized disease, compared with 38 months (95% CI: 5.6-70.3) in those with advanced disease. No statistically significant difference in overall survival (OS) was observed between *BRCA1 *and *BRCA2 *carriers (median OS: 116 months (95% CI: 63.6-168.4) for *BRCA1*; median not reached for *BRCA2*; log-rank p=0.408). These comparisons should be interpreted cautiously given the limited number of events.

Patients with *BRCA1/2* gPV and non-prostate urological cancers

We assessed 11 patients from 10 families with non-prostate urological cancer and *BRCA1/2 *gPV. The affected gene, identified pathogenic variant, histology, sex, age at diagnosis, personal history of cancer, and family history of cancer are summarized in Table 2.

**Table 2:** Other urological non-prostate cancer in BRCA1/2 carriers Age at diagnosis in years. F: female, M: male

Cancer site	Gene	Variant	Age at diagnosis	Sex	Histology/location	Personal history of other cancers	Familial history
Renal cancer	*BRCA1*	c.3331_3334del	43	F	Clear cell renal cancer	Breast	Breast
	*BRCA2*	c.7738C>T	76	F	Clear cell renal cancer	Breast	Renal, urothelial, duodenal cancer
		IVS7-3C>G	47	F	Clear cell renal cancer	Breast	Breast, gastric, gynecological cancer (fallopian)
		c.2808_2811del	65	F	Clear cell renal cancer	Breast	Gastric
Urothelial cancer	*BRCA1*	c.5266dup	87	M	Non-muscle-invasive bladder carcinoma	Prostate	Melanoma, ovarian
	*BRCA2*	c.9097dup	71	M	Non-muscle-invasive bladder carcinoma	Breast cancer, prostate	Breast, esophageal
		c.6633_6637del	60	F	Non-muscle-invasive bladder carcinoma	0	Breast, endometrial, ovarian
		c.7738C>T	65	M	Clear cell bladder carcinoma with neuroendocrine differentiation (stage IV)	0	Renal, urothelial, duodenal cancer
		c.156_157insAlu	77	M	Non-muscle-invasive bladder carcinoma	Biliary tract carcinoma, prostate	Breast
		c.156_157insAlu	57	M	High-grade muscle-invasive carcinoma of the ureter	Larynx	Breast
		c.156_157insAlu	71	M	Non-muscle-invasive bladder carcinoma	Breast, prostate	Gastric, pancreas, breast

The median age at diagnosis was 56 years (IQR 45-70.5) for patients with renal cancer and 71 years (IQR 62.5-74) for patients with urothelial cancer.

All patients with renal cancer were female and had a personal history of breast cancer, with tumors exhibiting clear cell histology. One patient carried a *BRCA1 *gPV and three carried *BRCA2 *gPV. None of the patients had a smoking history; smoking status was unavailable for one patient. All patients were treated surgically. The *BRCA1 *carrier reported a family history limited to breast cancer, whereas *BRCA2 *carriers reported family histories including other malignancies, such as gastric, renal, and duodenal cancers.

The median follow-up was 7.5 years (IQR 5.0-23.5), and mOS was 10 years (95% CI: 2.5-17.4).

Among patients with urothelial cancer, one patient carried a *BRCA1 *gPV, and six carried a *BRCA2* gPV. The majority presented with non-muscle-invasive bladder carcinoma (71.4%) except two *BRCA2 *carriers who had more advanced disease, including one case of clear cell bladder carcinoma with neuroendocrine differentiation (stage IV) and one case of high-grade muscle-invasive carcinoma of the ureter. Most patients were male (85.7%), with a single female patient who was a *BRCA2 *carrier. 

All patients diagnosed with non-muscle-invasive bladder carcinoma, except one, had a personal history of other malignancies. The *BRCA1 *carrier had a history of prostate cancer, which was also reported in three *BRCA2 *carriers. Additionally, *BRCA2 *carriers had histories of other cancers, including breast, biliary tract, and head and neck malignancies. Notably, all patients with a history of prostate cancer were over 70 years of age. Among the six *BRCA2 *gPV patients, three carried the same germline variant (c.156_157insAlu).

71.4% of *BRCA2 *gPV carriers reported a family history of breast cancer, and several also had relatives affected by other malignancies, including digestive tract cancers. Among *BRCA1 *carriers, family history was positive only for melanoma and ovarian cancer.

Smoking history was available for six patients, of whom four reported a history of smoking and two had never smoked. All patients underwent transurethral resection of the bladder (TURB), except one patient who was treated with surgery and another who was stage IV at diagnosis. The median follow-up was 26 months (IQR 14-51). mOS was reached.

No patient was diagnosed with testicular cancer and *BRCA1/2 *gPV.

*CHEK2 *carriers

One hundred and thirty-two families were included, of which six (4.5%) families had patients with a diagnosis of urological cancer and a *CHEK2* gPV. One patient had a renal cancer diagnosis, and five patients had a prostate cancer diagnosis.

Clinicopathological and genetic characteristics, including the identified pathogenic variant, histology or Gleason score, sex, age at diagnosis, and personal and family cancer history, are presented in Table 3.

**Table 3:** Urological cancer in CHEK2 carriers Age at diagnosis in years. Gleason score based on the 2005 International Society of Urological Pathology (ISUP) Consensus Conference on Gleason Grading of Prostatic Carcinoma [19]. F: female; M: male

Cancer site	Variant	Age at diagnosis	Sex	Histology/location/Gleason score if applicable	Personal history of other cancers	Familial history
Renal cancer	c.319+2T>A	43	F	Clear cell renal cancer	Breast	Breast
Prostate cancer	c.1100del	61	M	G6 (3+3)	Breast	Breast, ovarian, stomach
	c.1100del	74	M	G7 (4+3)	0	Colorectal
	c.467dup	63	M	G7 (3+4)	Papillary thyroid carcinoma	Thyroid, breast
	c.1036C>T	66	M	G7 (4+3)	0	Breast, gastric, pancreas
	c.349A>G	64	M	G6 (3+3)	0	Breast, prostate, pancreas

The median age at cancer diagnosis was 64 years (IQR 62-70) for patients with prostate cancer. Three patients with prostate cancer reported a family history of breast cancer, and just one disclosed a family history of prostate cancer.

The renal cancer patient was female and underwent surgical treatment, with no evidence of disease at the last follow-up. The majority of patients with prostate cancer presented with a Gleason score of 7 (either 3+4 or 4+3), whereas fewer cases exhibited a Gleason score of 6 or 8. All patients had localized disease at the time of diagnosis, and no disease progression was observed during follow-up. Regarding treatment, one patient underwent radical prostatectomy; three received radiotherapy followed by androgen deprivation therapy; two were treated with radiotherapy alone; and one patient received androgen deprivation therapy exclusively.

The median follow-up was 7 years (IQR 3.5-13.5). In terms of survival data, mOS was not achieved in renal or prostate cancer patients, and no patient died of cancer-specific causes. No patient was diagnosed with testicular or urothelial cancer and *CHEK2 *gPV.

## Discussion

This retrospective descriptive study characterizes urological cancers occurring in carriers of gPVs in *BRCA1*, *BRCA2*, and *CHEK2 *followed at a Portuguese Hereditary Cancer Risk Clinic.

Among the 968 families assessed, 47 families included patients with urological cancer who carried a gPV in one of the previously described genes, comprising a total of 50 affected individuals. As expected, most patients carried *BRCA2 *gPVs rather than *BRCA1 *gPVs, consistent with the stronger and more consistently reported association between *BRCA2 *and prostate cancer risk [11]. This finding may also reflect the presence of a Portuguese founder *BRCA2 *gPV present in nine patients with urological cancer. Although the absolute number of affected patients was limited, the analysis was based on a large pool of families followed longitudinally, supporting the representativeness of the observed spectrum.

*BRCA1/2 *carriers and prostate cancer

In our cohort, prostate cancer represented the most frequent urological malignancy among *BRCA1/2* carriers. The median age at diagnosis among *BRCA2* carriers (61 years) was lower than that previously reported for prostate cancer in the general Portuguese population (68 years) [20], whereas *BRCA1 *carriers were diagnosed at a later median age (75 years). Given the small sample size, this difference should be interpreted cautiously and may reflect sporadic variation rather than a true biological effect.

As expected, most patients presented with localized disease and intermediate-grade tumors; however, a subset exhibited high-grade or metastatic disease at diagnosis and had a *BRCA2 *gPV, consistent with prior reports linking *BRCA2 g*PV to more aggressive prostate cancer phenotypes [21]. *BRCA2 *carriers also showed a more diverse personal cancer history, with breast cancer being the most frequently reported additional malignancy, an association not found among *BRCA1 *carriers, and included five patients with a history of more than one additional malignancy.

In family history, breast cancer was the most frequently reported malignancy in both *BRCA1 *(71%) and *BRCA2 *(74%) carriers, followed by prostate cancer (42% in *BRCA1 *and 34% in *BRCA2*). In addition, *BRCA2 *families exhibited a broader spectrum of associated malignancies, including pancreatic, colorectal, and gastric cancers, as well as melanoma.

With respect to *BRCA *gPV, previous studies show no genotype-phenotype correlation for specific *BRCA1 *variants, but have identified a prostate cancer cluster region in the 3′ end of *BRCA2 *(c.7914-3′), with pathogenic variants in this region associated with increased prostate cancer risk [22,23]. In our cohort, three pathogenic *BRCA2 *variants (c.8488-1G>A, c.9097dup, and c.9815del) mapped to this region. These observations indicate that variants located within previously described prostate cancer cluster regions are present in our population; however, formal risk analyses were not performed and are not possible given the descriptive nature of this study.

Historically, *BRCA1/2* gPVs in metastatic prostate cancer have been associated with poorer prognosis [24]. In our series, the median progression-free survival among patients diagnosed with stage IV disease was 14 months. These findings are descriptive only and should be interpreted with caution, given the limited number of metastatic cases, the retrospective design, and the lack of stratification according to castration sensitivity. Additionally, most patients were treated before the widespread introduction of PARP inhibitors in the metastatic setting in Portugal.

*BRCA1/2* carriers and non-prostate urological cancer

Although less frequent, renal and urothelial cancers were also identified among *BRCA1/2* carriers. All renal cancer cases occurred in women and were associated with a personal history of breast cancer. The median age at diagnosis for renal cancer was 56 years, which is slightly lower than the approximately 60 years previously reported in the general population in other studies [25,26].

In contrast, the median age at diagnosis of urothelial cancer was similar to that reported in other studies [27]. Most patients presented with non-muscle-invasive urothelial carcinoma of the bladder; however, those with invasive disease were two *BRCA2* carriers, including one patient diagnosed with stage IV disease and one case of high-grade muscle-invasive carcinoma of the ureter. To date, there is no clear evidence supporting an association between *BRCA2 *gPV and worse prognosis in urothelial bladder cancer and risk stratification. In our cohort, the limited number of cases precluded meaningful risk stratification or prognostic analysis.

Most patients with non-muscle-invasive bladder cancer had a personal history of other malignancies, which may reflect a surveillance bias due to closer medical follow-up. Notably, all patients with a history of prostate cancer were over 70 years of age at diagnosis.

Among *BRCA2 *carriers, three shared the same germline variant (c.156_157insAlu), the Portuguese founder variant, including one patient with invasive ureteral disease, although this observation cannot support a causal association.

We did not identify any patients carrying the *BRCA2 *c.9976A gPV, which has previously been associated with an increased risk of bladder cancer [13]. While tobacco exposure is a well-established risk factor for urothelial cancer, smoking history was not systematically available in our cohort, limiting conclusions regarding the contribution of environmental risk factors.

*CHEK2* carriers and urological cancer

In our series, 132 families with *CHEK2 *gPV were assessed, of which 4.5% included patients diagnosed with urological cancer. Prostate cancer was the most frequently observed malignancy, affecting six patients, while renal cancer was identified in one patient.

The median age at diagnosis for prostate cancer was diagnosed at an age comparable to that reported in published series [20].

Both *CHEK2*-associated prostate and renal cancers were localized at diagnosis, and the prostate cancers were predominantly of favorable or intermediate risk. During follow-up, outcomes were favorable, with no cancer-specific deaths observed; however, these findings should be interpreted cautiously given the small sample size and limited follow-up duration.

Among *CHEK2* gPV, a moderate penetrance gene, pathogenic truncating variants (PTVs), most notably c.1100del, have been more consistently associated with an increased overall cancer risk, whereas pathogenic missense variants (PMVs) appear to confer more variable risk estimates [16]. The c.1100del variant has been associated with prostate cancer risk, although it has not been clearly linked to the familial clustering of prostate cancer [28]. In our cohort, two patients carried this variant, neither of whom reported a family history of prostate cancer.

The association between *CHEK2 *PV and renal cancer remains less well-established. While some studies suggest a possible increased risk, available data are limited, and further evidence is required to determine the clinical relevance of this association and to assess the cost-effectiveness of targeted screening strategies in *CHEK2 *carriers [29].

Although previous studies have suggested an association between *CHEK2* PV and an increased risk of testicular germ cell tumors, no cases of testicular cancer were identified among *CHEK2 *carriers in our cohort [30]. This may reflect the rarity of this malignancy, limited cohort size, or population-specific differences and underscores the need for larger studies to clarify this potential association.

Limitations

This study has limitations inherent to its retrospective design. Clinical and familial data were collected from both paper and electronic medical records, resulting in incomplete documentation for some variables, particularly smoking history and cancer diagnoses among extended family members.

As in many familial cancer studies, confirmation of reported diagnoses in relatives was not always possible due to loss of contact, death, or referral bias, which may have led to an underestimation of the true prevalence of urological cancers within families.

Survival analyses were also constrained by small sample sizes and by heterogeneity in treatment approaches across the long study period. Interpretation of OS is further limited by the advanced age of many patients and by the absence of cancer‑specific mortality data. In addition, OS was calculated from the date of diagnosis rather than from the start of treatment, which may influence survival estimates and complicate comparisons with other published series. The small number of advanced-stage cases and the absence of stratification by disease state or treatment era substantially limit the validity of the survival analyses and prevent definitive conclusions regarding survival differences. These factors reinforce the secondary nature of the survival outcomes.

Missing data in smoking status limits the possibility of drawing conclusions regarding this variable.

Finally, the study population was derived from a specialized Familial Risk Clinic, which introduces potential selection and surveillance bias, as these individuals may undergo more intensive screening and follow-up than the general population. Moreover, the absence of a comparator group precludes the direct estimation of relative risk and limits the ability to draw causal inferences.

## Conclusions

Despite these limitations, this study underscores the importance of continued investigation into the spectrum of urological cancers associated with gPVs in *BRCA1*, *BRCA2,* and *CHEK2*. Descriptive studies such as this remain essential to improving the characterization of cancer patients and clinical presentation and outcomes in genetically predisposed populations. 

This is particularly relevant in Portugal, where a founder pathogenic variant in *BRCA2 *is relatively prevalent and population‑specific data remain limited. Recurrent patterns of urological and *BRCA*‑associated malignancies within families may support the earlier identification of at‑risk individuals, timely referral for genetic counselling, and implementation of tailored surveillance strategies for carriers and their relatives.
